# Ovarian cancer: density equalizing mapping of the global research architecture

**DOI:** 10.1186/s12942-016-0076-2

**Published:** 2017-01-13

**Authors:** Dörthe Brüggmann, Katharina Pulch, Doris Klingelhöfer, Celeste Leigh Pearce, David A. Groneberg

**Affiliations:** 1 Department of Obstetrics and Gynecology, Keck School of Medicine of USC, Los Angeles, CA USA; 2 Department of Female Health and Preventive Medicine, Institute of Occupational Medicine, Social Medicine and Environmental Medicine, Goethe-University, Theodor-Stern Kai 7, 60590 Frankfurt, Germany; 3 Department of Epidemiology, School of Public Health, University of Michigan, Ann Arbor, MI USA

**Keywords:** Ovarian carcinoma, Density equalizing mapping, Socio-economic analysis

## Abstract

**Background:**

Despite its impact on female health worldwide, no efforts have been made to depict the global architecture of ovarian cancer research and to understand the trends in the related literature. Hence, it was the objective of this study to assess the global scientific performance chronologically, geographically and in regards to economic benchmarks using bibliometric tools and density equalizing map projections.

**Methods:**

The NewQIS platform was employed to identify all ovarian cancer related articles published in the Web of Science since 1900. The items were analyzed regarding quantitative aspects (e.g. publication date, country of origin) and parameters describing the recognition of the work by the scientific community (e.g. citation rates).

**Results:**

23,378 articles on ovarian cancer were analyzed. The USA had the highest activity of ovarian cancer research with a total of n = 9312 ovarian cancer-specific publications, followed by the UK (n = 1900), China (n = 1813), Germany (n = 1717) and Japan (n = 1673). Ovarian cancer-specific country h-index also showed a leading position of the USA with an h-index (HI) of 207, followed by the UK (HI = 122), Canada (HI = 99), Italy (HI = 97), Germany (HI = 84), and Japan (HI = 81). In the socio-economic analysis, the USA were ranked first with an average of 175.6 ovarian cancer-related publications per GDP per capita in 1000 US-$, followed by Italy with an index level of 46.85, the UK with 45.48, and Japan with 43.3. Overall, the USA and Western European nations, China and Japan constituted the scientific power players publishing the majority of highly cited ovarian cancer-related articles and dominated international collaborative efforts. African, Asian and South American countries played almost no visible role in the scientific community.

**Conclusions:**

The quantity and scientific recognition of publications related to ovarian cancer are continuously increasing. The research endeavors in the field are concentrated in high-income countries with no involvement of lower-resource nations. Hence, worldwide collaborative efforts with the aim to exchange epidemiologic data, resources and knowledge have to be strengthened in the future to successfully alleviate the global burden related to ovarian cancer.

**Electronic supplementary material:**

The online version of this article (doi:10.1186/s12942-016-0076-2) contains supplementary material, which is available to authorized users.

## Background

Ovarian cancer is the most lethal gynecological tumor in high income-countries; it represents the seventh-most common female cancer worldwide [1, 2]. In the United States, approximately 22,000 new ovarian cancer cases are diagnosed annually, 14,200 related deaths occur each year [3]. The majority of invasive ovarian malignancies originate from epithelial cells. Each histotype—high-grade serous, low-grade serous, mucinous, clear cell and endometrioid—exhibits distinct clinical and pathological characteristics [4].

During the last three decades, multiple breakthrough discoveries have been reported in the field: for the last 10 years it has been accepted that two types of epithelial ovarian cancers exist [1, 5]. Type I tumors include low-grade serous, endometrioid and clear cell histologies [6, 7]. The association of Type I cancers with endometriosis was found in 2012. This benign condition increases the risk of low-grade serous and endometrioid cancers by approximately twofold, for clear cell subtypes by threefold [1, 8]. Also, *ARID1A* gene mutations were described for endometriosis-associated endometrioid and clear cell cancers [9]. Type II high-grade serous carcinomas are the most common ovarian malignancies. In 2006, Medeiros et al. presumed their origin from the fimbriae of the fallopian tube [10]. In the last years, the identification of relevant somatic and germline mutations gained relevance as a first step towards screening strategies and novel targeted therapies: *KRAS*, *BRAF*, *ERBB2*, *CTNNB1*, *PTEN*, *PIK3CA*, *ARID1A*, *PPP2R1A*, and *BCL2* mutations were found in Type I carcinomas. 96% of high-grade serous Type II tumors had *TP53* mutations [1, 5, 11]. In 1994 and 1995, *BRCA 1*/*2* mutations were described in hereditary Type II cancers; since then they have gained importance for clinical risk prediction and patient counseling [12, 13].

The volume of scientific literature in oncology increased rapidly during the last 50 years [14]. Systematic evaluation of research output is necessary to guide individual reading, to plan research activities according to shortcomings and to quantify individual and collaborative productivity on national and international level. These assessments play an integral role in career decisions, allocation of grant funding and prioritizing research resources [14]. Scientometric methods provide the standardized analysis of journal articles in reference to their content and citations describing developments in origin and dissemination of published data. Specific to ovarian cancer, no systematic evaluation of the global scientific output is available to date, and no efforts have been made to understand trends in the related literature. Therefore, the topic of ovarian cancer was elected by the New Quality and Quantity Indices in Science (NewQIS) project [15] for a scientometric in-depth analysis. The study objectives included (1) the assessment of the worldwide publication output regarding quantitative aspects, parameters describing the recognition within the scientific community (e.g. citation rates) and research networks as well as (2) the evaluation of the country-specific productivity related to socio-economic variables. Also, we identified the leading journals publishing in the field and the most recognized articles since 1900.

## Methods

### NewQIS study

We employed the established NewQIS platform [15, 16] to conduct this study. The NewQIS platform was developed in 2009 as a multidisciplinary project involving scientists from different backgrounds such as engineering, computer sciences and medicine and numerous studies were published so far using the platform [17–32]. It constitutes a novel tool that was designed for the objective, precise and reliable scientometric analyses of research productivity based on validated protocols. Benefits of the platform include the efficient and standardized investigation of the scientific progress chronologically and geographically, the visualization of the results in expressive global maps via density equalizing map projections (DEMP), as well as unique evaluation tools deciphering national and international scientific relations and gender distribution among authors.

### Data source

We used an index database of the Web of Science (WoS core collection, Thomson Scientific) and analyzed the total research productivity by quantification of ovarian cancer-specific publications. Parameters describing the articles’ recognition by the scientific community were assessed based on the number of related citations, i.e. h-indices and citation rates.

The WoS was selected as data source because of its unique Citation Report function allowing the extraction of citation performance parameters [33]. We refrained from extracting data from other platforms such as Google Scholar or Scopus due to the lack of data congruence in these three databases hampering triangulating, comparing and integrating data related to ovarian cancer research since 1900 [34].

### Search strategy

We conducted a “title” search for the time period of 1900 (01-01) to 2014 (31-12). The search term [“(*ovarian OR ovary*) *AND* (*cancer OR neoplasm OR carcinoma*)”] was used. The year 2015 was excluded to avoid incomplete data acquisition at the time the study was performed. We used the filter option “document type” to restrict our search to “original articles” as described previously [15].

### Data analysis and categorization

Articles were saved in a plain text format using the download application provided by the WoS. All related metadata were collected in an interim database and, analyzed according to the following criteria: originating country, language, citations, cited references, authors, journal, year published and subject categories. The subject categories represent standard categories assigned to every publication by the Journal Citation Reports (provided by the Thompson Reuters/Institute of Scientific Information) during the publication process. We computed the country-specific modified h-index (HI) and the citation rate (CR, number of all citations per total ovarian cancer publication volume). In 2005, the HI was developed by Jorge Hirsch to assess the recognition of an author’s research performance in the scientific community [35]. In our study, this proxy measure was adapted to evaluate the productivity of single countries in ovarian cancer research and therefore termed “modified HI”. Also, a glossary was added in the Additional file 1 describing important terms used in this manuscript.

### Density equalizing map projections (DEMP)

DEMP visualize benchmarking processes by the creation of anamorphic world maps. After the transfer of the metadata to excel charts and parameter analysis, DEMP were calculated based on the algorithms of Gastner and Newman. Therefor, the territories of countries publishing ovarian cancer research were resized in proportion to the selected criteria (i.e. the total number country-specific articles) [36].

### Socio-economic analysis

In order to quantify country-specific contributions to ovarian cancer research in regards to their economic resources and manpower, we evaluated research productivity in relation (1) to the gross domestic product (GDP) per capita, (2) to the total economic power index GDP per 1000 billion US-$ and (3) to the population size. Economic facts were obtained from the *World Economic Outlook Database of the International Monetary Fund* of 2014 [37]. Only countries with a minimum of 50 ovarian cancer publications were included. We also collected the absolute numbers of ovarian cancer incidence and the crude rate (defined as the new cancer cases diagnosed in a specific year per 100,000 persons at risk) of the 25 countries that have published more than 100 ovarian cancer items during the investigated time span. The data reflect the incidence of ovarian cancer in 2012 and were obtained from http://globocan.iarc.fr/Pages/summary_table_pop_sel.aspx. Based on these numbers we calculated the ratio of country-specific articles per each new ovarian cancer case.

### Analysis of ovarian cancer research collaborations

To determine research collaborations from a global viewpoint, affiliations of authors were analyzed and chart diagrams were computed as previously described [38]. We defined an article as “collaborative” if at least two authors, who work in different countries as stated in the affiliations, contributed to the work. Publications with shared authorship were counted one time only (independent of the number of authors from the same country defined in the affiliations) towards the complete count of joint publications this specific country is involved in. For example, when 10 publications were analysed of which eight were affiliated with the USA, five with the UK and three items were joint publications, these were counted as 3 out of 8 for the USA and 3 out of 5 for the UK. Also, we related the total count of collaborative items to the overall number of publications for each investigated country. For example, 2240 items were published by US-American authors in a joint effort with other countries. These were related to the overall scientific productivity of the USA represented by 9312 items (24%). 747 collaborative publications were identified for the UK; these accounted for 39% out of 1900 items. In Fig. 3, vectors represent the productivity of collaborations for each pair of countries. These are proportional in line width and shade of grey to the number of collaborations.

## Results

### General parameters

**Fig. 1:**
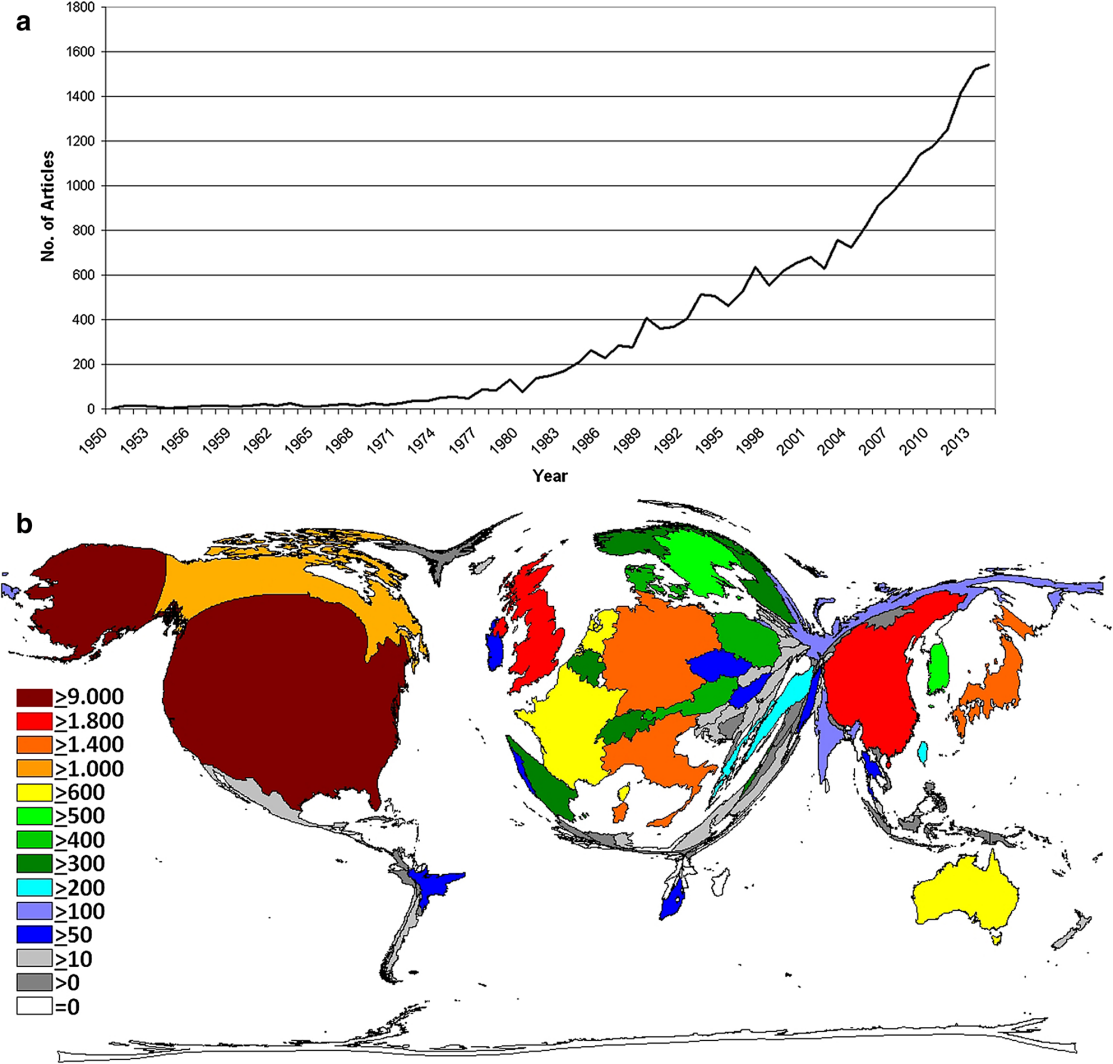
Publication output. **a** Number of published items per year. **b** Density equalizing map of the global ovarian cancer research activity between 1900 and 2014. *Colors* and *territorial sizes* indicate numbers of ovarian cancer publications per country

In 115 years, a total of 23,378 original articles were published in the WoS. The publication activities increased continuously throughout the decades: Until the 1950s we identified up to 10 articles each year; this number increased to more than 100 publications/year from 1979 onwards and doubled after 1984. In the next decade, the productivity increased to more than 500 annual items and doubled again after 2008. In 2014, 1540 articles were published (Fig. 1a). The number of participating authors per publication increased from 2.5 authors in 1972 to 8.18 authors per ovarian cancer-related article in 2014.

### Country-specific analysis

A total of 99 countries participated in the publication of all articles. The majority of publication volume originated from a small number of countries: The United States of America (USA) was the most productive with 9312 ovarian cancer-specific articles. It was followed by the United Kingdom (UK, 1900 articles), China (1813 articles), Germany (1717 articles), Japan (1673 articles) and Italy (1672 articles). Hence, DEMP analysis demonstrated a distorted world map with the main focus on North America and Western Europe and a prominent China and Japan (Fig. 1b). Asian, South American and African countries occupied only minimal areas on the cartogram.

## Citation analysis

**Fig. 2:**
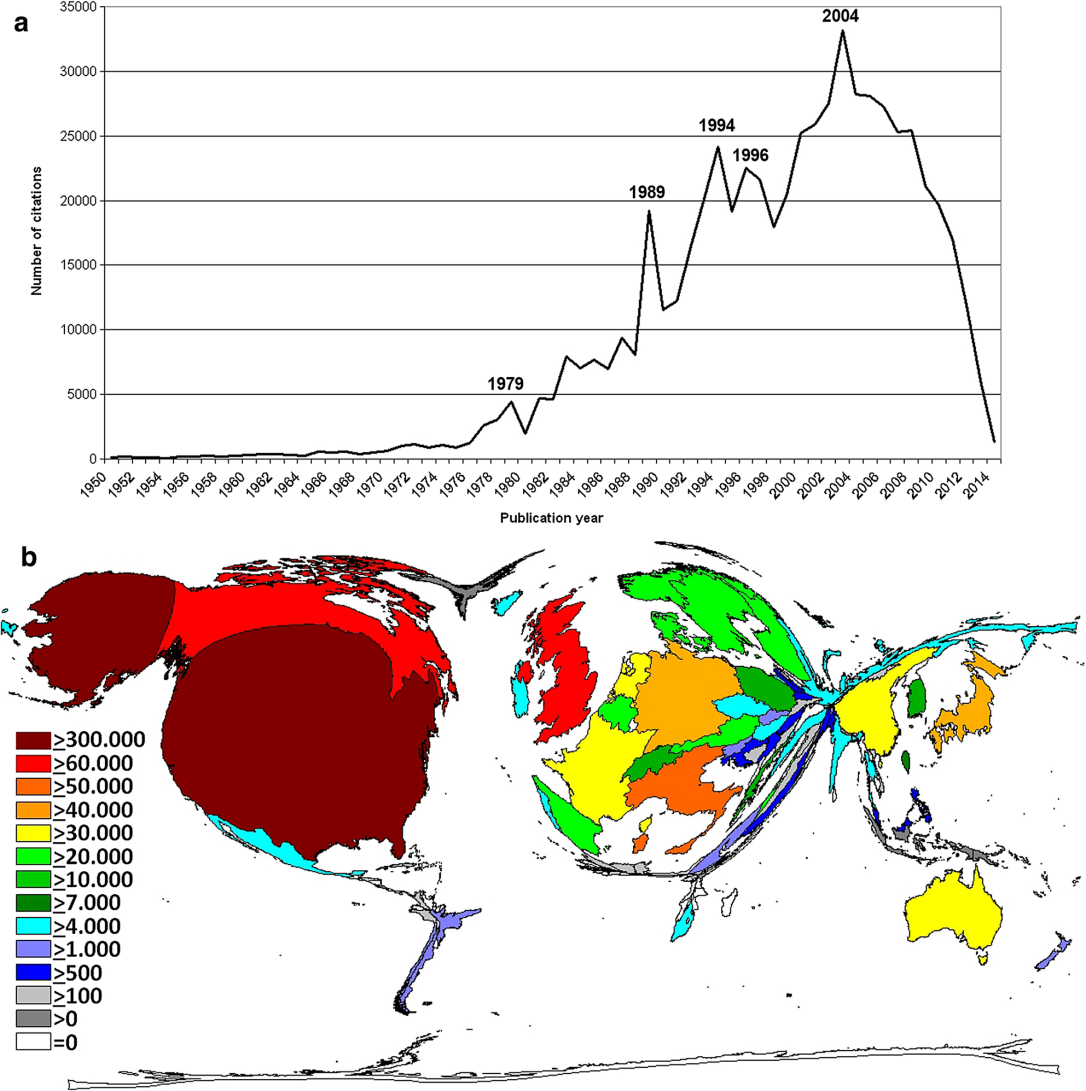
Citation analysis. **a** Number of citations between 1950 and 2014. **b** Density equalizing map of the number of citations. *Colors* and *territorial sizes* indicate numbers of citations per country

The citation count of yearly published articles showed a course similar to the annual publication activity: After a very modest increase until 1974 the citations increased steadily with peaks in 1979, 1989, 1994, 1996, 2004. After 2005, we documented a steep decline in citation numbers until 2014 with the exception of a small plateau in 2008 (Fig. 2a).

Country-specific citation analysis indicated a leading position of the USA with 354,891 citations (41.2% of all citations). It was followed by the UK (71,562 citations), Canada (55,964 citations), Italy (49,422 citations), Japan (35,995 citations), and Germany (34,278 citations) (Fig. 2b). In contrast to publication activities, China dropped from third to position 10 when citations were quantified.

The USA dominated the country-specific HI analysis (HI of 207), and was followed by the UK (HI = 122), Canada (HI = 99), Italy (HI = 97), Germany (HI = 84), and Japan (HI = 81) (Table 1). Regarding the citation rate (CR) of countries with a minimum of 30 articles published on ovarian cancer, we identified Canada (CR = 43.52) in the leading position. Then Finland (CR = 39.17), Hungary (CR = 38.81), the USA (CR = 38.11) and the UK (CR = 37.66) were followed by the Western European countries Belgium (CR = 36.92), Sweden (CR = 36.61), the Netherlands (CR = 34.03), Norway (CR = 31.8), Italy (CR = 29.56) and France (CR = 23.79). China dropped to a CR of 11.05 (Table 2).

**Table 1:** Modified h-indices

Rank	Country	h-index
1	United States	207
2	United Kingdom	122
3	Canada	99
4	Italy	97
5	Germany	84
6	Japan	81
7	Netherlands	78
8	Australia	74
9	France	70
10	Sweden	68
11	Belgium	58
12	China	57
13	Finland	55
14	Denmark	54
15	Norway	54
16	Israel	52
17	Austria	51
18	Spain	49
19	Switzerland	48
20	South Korea	45
21	Greece	42
22	Poland	40
23	Taiwan	37
24	Turkey	23
25	India	22
26	Ireland	22
27	South Africa	20
28	Hungary	20
29	Portugal	20
30	Czech Republic	19
31	Russia	18
32	Thailand	18
33	Singapore	18
34	Iceland	18
35	Brazil	16
36	New Zealand	15
37	Mexico	14
38	Slovakia	14
39	Slovenia	13
40	Chile	13
41	Croatia	12
42	Egypt	12
43	Malaysia	12
44	Iran	10
45	Saudi Arabia	10
46	Argentina	10
47	Belarus	10
48	Serbia	9
49	Romania	8
50	Pakistan	8
51	Lithuania	8
52	Bulgaria	7
53	Latvia	6
54	Ukraine	4
55	Tunisia	3

The table summarizes the h-indices related to research on ovarian cancer and published by the countries investigated

**Table 2:** Ovarian cancer-specific citation rates

Rank	Country	Citation rate
1	Canada	43.52
2	Finland	39.17
3	Hungary	38.81
4	United States	38.11
5	United Kingdom	37.66
6	Belgium	36.92
7	Sweden	36.61
8	Netherlands	34.03
9	Norway	31.80
10	Australia	30.55
11	Italy	29.56
12	Portugal	29.44
13	Switzerland	29.43
14	Spain	28.76
15	Denmark	28.53
16	Ireland	28.51
17	Mexico	27.16
18	Israel	25.96
19	Greece	24.91
20	France	23.79
21	Austria	22.58
22	Slovenia	22.47
23	South Africa	22.35
24	Japan	21.52
25	New Zealand	20.66
26	Germany	19.96
27	Poland	19.33
28	Taiwan	18.17
29	Thailand	17.85
30	Czech Republic	17.16
31	Slovakia	16.69
32	South Korea	15.30
33	Egypt	13.81
34	Singapore	13.03
35	India	12.65
36	Malaysia	12.31
37	Brazil	11.90
38	China	11.05
39	Romania	10.50
40	Saudi Arabia	9.30
41	Croatia	8.82
42	Turkey	8.59
43	Russia	7.55
44	Iran	4.34
45	Serbia	4.14

The table summarizes the citation rates related to ovarian cancer research and published by investigated countries with a minimum of 30 publications

### Socio-economic analysis of ovarian cancer research

When the country-specific publications were related to the gross domestic product (GDP) per capita, the USA was ranked first with an average of 169.9 ovarian cancer-related publications per GDP per capita in 1000 US-$ (Q1). The USA was followed by China as the first middle-income country in the ranking (Q1: 140.5), the UK (Q1: 50.4), Italy (Q1: 48.5) and Japan (Q1: 44.3) (Table 3).

**Table 3:** Socio-economic analysis of ovarian cancer research of the most active countries in ovarian cancer research. *Source* for GDP (Currency in 1000 Billion US Dollars) and GDP per capita (Currency in 1000 US Dollars) in 2014 was the World Economic Outlook Database of the International Monetary Fund of 2014. (Threshold: 50 ovarian cancer-specific publications)

Country	Rank	Number of articles	GDP (in 1000 Bill. US$)	Articles/GDP (1000 Bill. US$)	Rank ratio (Articles/GDP in economic group)	GDP per capita (in US$)	Articles/GDP per capita (in 1000 US$)	Rank ratio (Articles/GDP per capita in economic group)
USA	1.	9312	17.420	534.6	HIG 14	54,800	169.9	HIG 1
China	2.	1813	10.360	175.0	MIG 4	12,900	140.5	MIG 1
UK	3.	1900	2.848	667.1	HIG 12	37,700	50.4	HIG 2
Italy	4.	1672	2.129	785.3	HIG 9	34,500	48.5	HIG 3
Japan	5.	1673	4.770	350.7	HIG 22	37,800	44.3	HIG 4
Germany	6.	1717	3.820	449.5	HIG 18	44,700	38.4	HIG 5
Canada	7.	1286	1.794	716.8	HIG 11	44,500	28.9	HIG 6
India	8.	150	2.048	73.2	MIG 6	5800	25.9	MIG 2
France	9.	878	2.902	302.5	HIG 24	40,400	21.7	HIG 7
Poland	10.	458	0.552	829.4	HIG 8	24,400	18.8	HIG 8
Australia	11.	742	1.483	500.3	HIG 16	46,000	16.1	HIG 9
Netherlands	12.	762	0.880	865.5	HIG 7	47,400	16.1	HIG 10
South Korea	13.	561	1.410	397.9	HIG 20	35,400	15.8	HIG 11
Turkey	14.	290	0.813	356.6	MIG 1	19,600	14.8	MIG 3
Sweden	15.	522	0.559	933.6	HIG 6	44,700	11.7	HIG 12
Israel	16.	388	0.305	1272.1	HIG 2	33,400	11.6	HIG 13
Greece	17.	295	0.246	1197.2	HIG 4	25,800	11.4	HIG 14
Spain	18.	370	1.400	264.3	HIG 25	33,000	11.2	HIG 15
Denmark	19.	449	0.347	1293.2	HIG 1	44,300	10.1	HIG 16
Austria	20.	450	0.436	1031.9	HIG 5	45,400	9.9	HIG 17
Finland	21.	337	0.276	1219.7	HIG 3	40,500	8.3	HIG 18
Belgium	22.	332	0.528	629.0	HIG 13	41,700	8.0	HIG 19
Russia	23.	166	2.057	80.7	HIG 28	24,800	6.7	HIG 20
Taiwan	24.	265	0.530	500.5	HIG 15	43,600	6.1	HIG 21
Norway	25.	391	0.512	764.3	HIG 10	65,900	5.9	HIG 22
South Africa	26.	71	0.341	208.1	MIG 2	12,700	5.6	MIG 4
Switzerland	27.	306	0.679	450.7	HIG 17	55,200	5.5	HIG 23
Thailand	28.	74	0.374	198.0	MIG 3	14,400	5.1	MIG 5
Brazil	29.	70	2.244	31.2	MIG 7	15,200	4.6	MIG 6
Iran	30.	62	0.403	154.0	MIG 5	16,500	3.8	MIG 7
Czech Republic	31.	74	0.206	359.9	HIG 21	28,400	2.6	HIG 24
Hungary	32.	57	0.130	439.5	HIG 19	24,300	2.3	HIG 25
Portugal	33.	54	0.228	236.6	HIG 26	26,300	2.1	HIG 26
Ireland	34.	75	0.246	305.1	HIG 23	46,800	1.6	HIG 27
Singapore	35.	70	0.308	227.3	HIG 27	81,300	0.9	HIG 28

For the total economic power index GDP, Denmark was positioned at the first place with a total of 1293.2 ovarian cancer-specific articles per 1000 billion US-$ GDP (Bio US-$ GDP, Q2), followed by Israel (Q2: 1272). Amongst the high-income countries, the UK ranked at position 12 (Q2: 667.1), followed by Belgium (Q2: 629) and the USA (Q2: 534.6). China (Q2: 157) occupied the 4th rank of the middle-income countries and the 32nd position of all countries with more than 50 ovarian cancer-specific articles (Table 3).

Denmark was positioned first when the ovarian cancer research output was related to population size. Here, 80.3 ovarian cancer-specific publications were authored per 1 million citizens. It was followed by Norway (77.9 publications/1 million citizens), Iceland (65.5 publications/1 million citizens), Finland (62.2 publications/1 million citizens) and Sweden at position 5 (54.8 publications/1 million citizens). Other productive countries were the USA (29.7 publications/1 million citizens), UK (29.8 publications/1 million citizens) and China (13.4 publications/1 million citizens).

Furthermore, Israel took the lead having published one article per newly diagnosed ovarian cancer case based on the incidence data of 2012. It was followed by Norway (0.94 articles per new ovarian cancer case), Denmark (0.83 articles per new ovarian cancer case), Sweden (0.79 articles per new ovarian cancer case), the Netherlands (0.74 articles per new ovarian cancer case) and Finland (0.74 articles per new ovarian cancer case). The USA was ranked 11th; countries such as China, Russia and India were ranked last amongst the 25 investigated nations (Table 4). A DEMP shows the absolute ovarian cancer incidence numbers of the 25 counties that have published more than 100 articles during the investigated time span (Additional file 2: Figure S1).

**Table 4:** The table depicts the absolute incidence numbers and the crude rate (defined as the new cancer cases diagnosed in a specific year per 100,000 persons at risk) of ovarian cancer of the 25 countries having published more than 100 related items and the ratio of country-specific articles per each new ovarian cancer case

Rank	Country	Article count	Incidence in 2012	Crude rate in 2012	Article/new case in 2012
1	Israel	388	380	9.8	1.02
2	Norway	391	418	16.9	0.94
3	Denmark	449	544	19.3	0.83
4	Sweden	522	659	13.8	0.79
5	Netherlands	762	1025	12.2	0.74
6	Finland	337	457	16.6	0.74
7	Austria	450	636	14.8	0.71
8	Australia	742	1424	12.4	0.52
9	Switzerland	306	621	15.8	0.49
10	Canada	1286	2648	15.2	0.49
11	United States	9312	20,874	13.1	0.45
12	Belgium	332	840	15.3	0.40
13	Greece	295	915	15.9	0.32
14	United Kingdom	1900	6692	21	0.28
15	Italy	1672	5911	19	0.28
16	Germany	1717	6673	16.1	0.26
17	South Korea	561	2349	9.8	0.24
18	France	878	4592	14.1	0.19
19	Japan	1673	8921	13.7	0.19
20	Turkey	290	2400	6.4	0.12
21	Spain	370	3236	13.7	0.11
22	Poland	458	4456	22.5	0.10
23	China	1813	34,575	5.3	0.05
24	Russia	166	13,373	17.4	0.01
25	India	150	26,834	4.4	0.01

The data reflect the incidence of ovarian cancer in 2012

### Publishing journals and landmark articles

1685 journals published ovarian cancer-related articles since 1900. The most prolific journal was “Gynecologic Oncology” with 2710 articles and a related citation rate (CR) of 23.91 followed by “International Journal of Gynecological Cancer” (968 articles/CR = 11.06) and “Cancer Research” (637 articles/CR = 80.81). We displayed the top 15 journals including number of articles, citations and CR (Additional file 3: Table S1, Additional file 4: Figure S2) and identified the ten most cited articles in the area of ovarian cancer research (Additional file 5: Table S2).

### Ovarian cancer subject area analysis

The leading subject categories of ovarian cancer research were “Oncology” with 13,649 publications cited 363,896 times, “Obstetrics & Gynecology” (6878 publications, 128,161 citations), and—following with a considerable gap—“Pathology” (1238 publications and 31,921 citations) (Additional file 6: Figure S3A). The areas “General & Internal Medicine” (35,221 citations) and “Genetics & Hereditary” (33,842 citations) showed a high CR relative to the total number of publications indicating a high impact of published work in the field.

We performed a subject area analysis for the ten most active countries in ovarian cancer research to identify their particular scientific focus: Up to 80% of all publications in nine of the ten countries were attributed to “Oncology” and “Obstetrics & Gynecology”. China published a high percentage of articles in “Research and Experimental Medicine”, “Biochemistry and Molecular Biology” as well as “Cell Biology”. Researchers from the UK, Australia, France and Canada focused on the area of “Genetics”. Japanese scientists dedicated a high percentage of their work to the subject category of “Pathology”. “General and Internal Medicine” was popular among researchers from France and the UK (Additional file 6: Figure S3B).

### International ovarian cancer collaborations

**Fig. 3:**
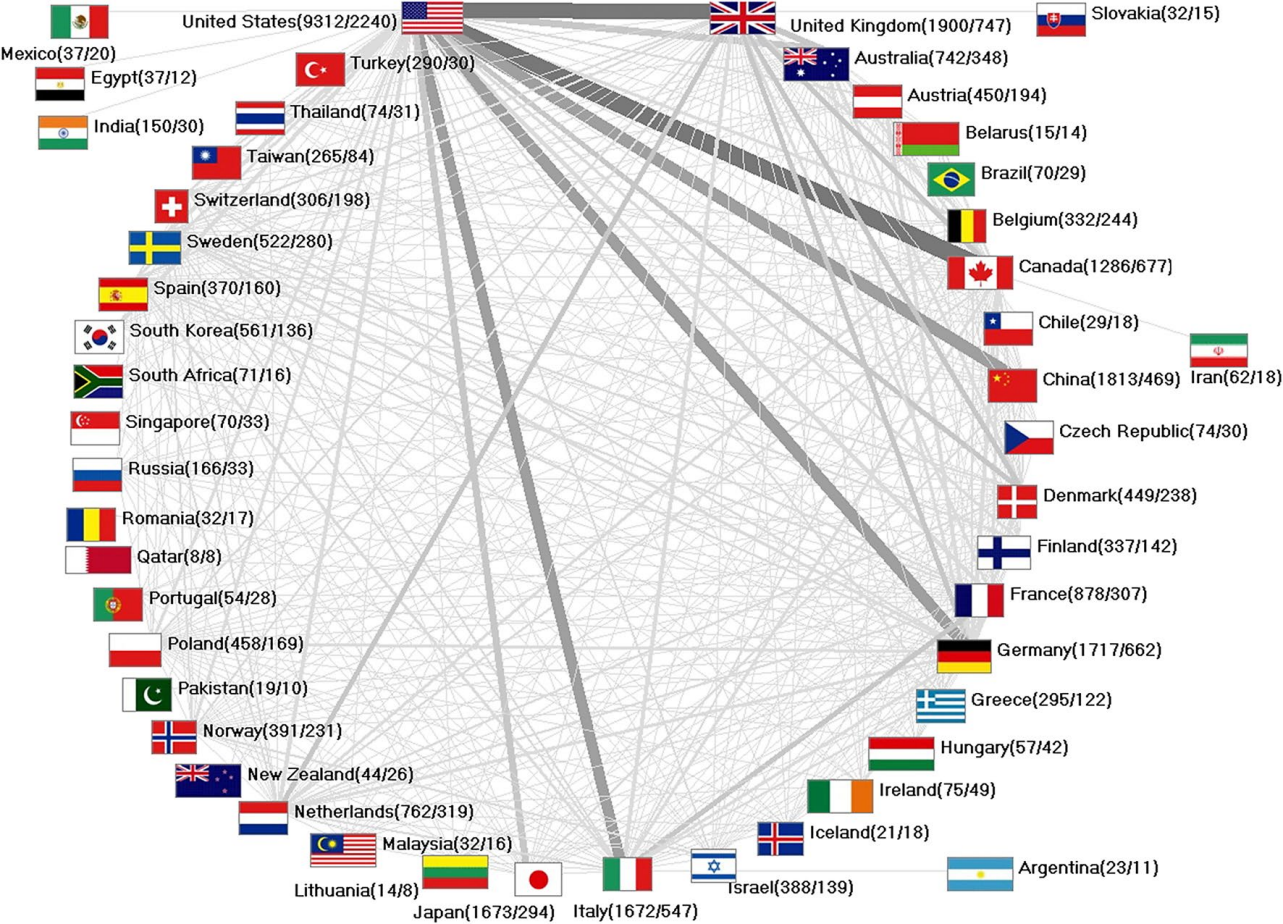
International ovarian cancer research collaborations. *Greyscale* and *bar thickness* indicate intensity of collaborations. *First ciphers in brackets* indicate total publication numbers. *Second ciphers* indicate number of collaborative publications. Threshold: 5 collaborations

We identified 3697 international collaborations publishing on ovarian cancer, 74% were bilateral (2733 items) and 15.4% trilateral co-operations (568 items). Joint research efforts were clearly dominated by scientists and institutions situated in the USA. US-American authors published 24% of all publications in co-operation with other countries, and collaborated with 13 different countries in total. The most active collaborations were established between the USA and Canada (433 collaborative papers), followed by US-American co-operations with the UK (385 papers), China (300 papers), Italy (291 papers) and Germany (284 papers) (Fig. 3).

## Discussion

During 115 years, a total of 23,378 original research articles were published in the WoS. The number of publications rose slowly until the seventies, when a steep and steady increase of research productivity started. This pattern is detected for most biomedical research as exemplified by studies on medical curare use or bacterial meningitis [39, 40]. From 1900 to 1950, only 139 articles related to ovarian cancer were part of the WoS database. This is attributed to the following: Overall research activities were lower since the recognition and funding of scientists were not predominantly determined by their productivity. In 1915, Japanese researchers could provoke cancer in an animal model for the first time. Since then pathogenetic mechanisms of cancer shifted into the scientific focus paving the way to today’s understanding of the disease [41]. English was not the common scientific language at this time. Hence, a considerable amount of non-English publications issued before 1950 is not represented in our analysis.

The late 1970s was an era when ovarian cancer-associated research gained increasing popularity (Fig. 1). Then, major scientific progress happened in the field as indicated by the first highly cited publication linking ovarian cancer and incessant ovulation [42–46]. The output grew dramatically in the nineties, which coincided with more landmark findings such as the discovery of the *BRCA* genes [47]. After 2008, annual research productivity increased to more than 1000 papers when new hypotheses regarding the origin of high-grade serous subtypes [10] and the association of ovarian cancers with *ARID1A* mutations and endometriosis were proposed [8, 9]. Also, publications assessing novel treatment strategies such as pathway inhibitor (e.g. PARP inhibitors) or antibody-based therapies were mainly released in the last 7 years.

Resembling the growing volume of published papers, the absolute citation count of ovarian cancer-related publications showed a steady increase until 2005 (Fig. 2a). Landmark papers (included in Additional file 5: Table S2) contributed to peaks in the graph: In 1979, Casagrande et al. [42] proposed the link between ovarian cancer and incessant ovulation. In 1989, two highly cited papers were published, which explored the pathogenetic relevance of HER2neu receptors and the efficacy of taxol as ovarian cancer treatment [48, 49]. In 1994 and 1995, the ovarian cancer susceptibility genes—BRCA 1 and 2—were identified. Related articles lead to citation peaks in 1994 and 1996 [12, 13]. A meta-analysis investigating the ovarian cancer risk of 8139 patients with BRCA1 and BRCA2 mutations was published in 2003 and associated with the citation peak in 2004 [50]. The decrease in citations after 2005 is linked to a delay of up to 8 years between publication and appropriate scientific recognition of an article represented by a maximum number of citations [51].

When country-specific ovarian cancer research productivity was analyzed, the leading position of the USA became evident. This finding aligns with a benchmarking study assessing the scientific output from 1961 to 2007 related to 22 organ systems. With 1,893,800 of 5,527,558 publications, the USA identified as the most productive nation [52]. The success of the USA points to its commitment to allocate major resources towards biomedical research, e.g. the NCI awarded $100.6 million ovarian cancer funding in 2003 (http://www.cancer.gov/research/progress/snapshots/ovarian). In our study, the USA was followed by the UK, China, Germany and Japan regarding research productivity. Glynn et al. [14] described a similar pattern for breast cancer, where the USA, the UK, Germany and Japan were also among the top five countries. China constitutes an exception: It ranked second for ovarian cancer research productivity but dropped to position 12 for breast cancer.

Citations indicate the relevance of a published item [14]. In our study, the USA, the UK and Canada dominated the ranking in term of citation counts, HI and CR. These results correspond to other studies in obstetrics and gynecology, e.g. on smoking and pregnancy. Here, the USA, the UK and Canada also achieved the highest modified HI of 128, 79 and 62 and the highest citation rates of 41.4, 8.6 and 5.3%, respectively [38]. In order to define the commitment of single countries in ovarian cancer research, we investigated scientific productivity in terms of socio-economic abilities and demonstrated two important features: First, the USA lost its leading position and other—mostly European—nations gained importance. Denmark, Norway, Iceland, Finland and Sweden ranked in the top five when article count was analyzed in relation to number of citizens. When the total number of articles was related to the total economic power, Israel, Iceland, Denmark and Finland were leading the field. Second, when we focused on countries with a large population and high total GDPs such as China, their relative contribution to the global research output remained small compared to the USA and Europe.

Taken together, the worldwide research architecture on ovarian cancer revealed that the USA and Western European nations, China and Japan constitute the scientific power players. They publish the majority of highly cited ovarian cancer-related articles and dominate international collaborative efforts. A strong dedication of single countries to ovarian cancer research is also indicated by the prominent position of European nations—e.g. the Scandinavian countries in particular—when research productivity was related to socio-economic benchmarks. These findings coincide with other benchmarking studies [52] and with the fact that the highest incidence rates of ovarian cancer (e.g. Northern and Western Europe with incidences of 13.3 and 11.3 per 100,000 person-years as well as Northern America with an incidence of 10.7 per 100,000 person-years) are found in areas with the greatest research productivity. This association underlines that the nations, which should prioritize ovarian cancer research to alleviate the burden among its female inhabitants, actually do so. Taking the ratio of articles per every new ovarian cancer case (based on the absolute incidence data of 2012) into account, we can demonstrate a similar picture. The Scandinavian countries and the Netherlands were among the leading nations. By contrast, Canada and the USA were ranked only in the middle field with a publication output of around one article per two new ovarian cancer cases. Although China had a low incidence rate of 3.2 per 100,000 person-years reported for 2002, secular epidemiological trends project increasing numbers for the future [53]. This might explain why China supports ovarian cancer research as indicated by the strong research productivity [54].

The public health burden of ovarian cancer is significant. No considerable improvements of survival rates or decrease in morbidity and mortality have been seen over the past decades. Hence, research activity needs to be fostered, and collaborative research efforts are crucial to tackle the challenges in the field. National and international networks are equipped to do this by sharing resources, facilities and ideas leading to landmark publications [55]. In our study, the USA was the most preferred nation for collaborations based on its outstanding financial support and scientific infrastructure. We identified the most productive co-operations between the USA, Canada as well as the UK. This finding is linked to the areal proximity and cultural/language similarities, which contribute to the high productivity and quality of research produced by each of these countries. Additionally, our observation of increasing author numbers per ovarian cancer publication reflects the development of strong research networks around the globe. The Ovarian Cancer Association Consortium (OCAC) serves as an example for highly prolific global networks. Founded in 2005, this multidisciplinary, international group published more than 60 high impact papers in the areas of genetics and epidemiology.

According to the International Agency for Research on Cancer in 2012, 58% of ovarian cancer cases occurred in less developed nations [56]. Countries with the highest incidence rates of ovarian cancer were Fiji (age-standardized rate per 100,000: 14.9), Latvia (age-standardized rate per 100,000: 14.2) and Bulgaria (age-standardized rate per 100,000: 14.0) [56]. Although these countries experience a significant burden due to the disease, they were underrepresented on our map of ovarian cancer research. We did not identify one article published by Fiji. Latvia published 10 articles cited 112 times and Bulgaria issued 10 articles cited 90 times. Fiji was not part of any collaborative network. Latvia participated in only five multinational collaborations and Bulgaria in eight bilateral collaborations. We want to point out the necessity—and almost the ethical responsibility—to include lower-resource countries with high incidence rates in scientific collaborations. Here epidemiological data, ideas and gained knowledge can be exchanged and benefit all participants.

To date, most ovarian cancer related articles were published in the subject categories of Oncology, Obstetrics/Gynecology as well as Pathology. This is not surprising. However, we found a shift in publication activity to areas such as Genetics and Internal Medicine. This development is linked to highly cited articles published by recently founded genetic-epidemiological consortia, i.e. OCAC or Ovarian Tumor Tissue Array Consortium. The increase in the subject category of Internal Medicine is explained by the growing number of high quality publications in leading journals such as the “New England Journal of Medicine”, “Nature” or “Lancet”, which are attributed to this category. Also, it reflects the growing interest of internal medicine physicians in the care of ovarian cancer patients.

In this study, we analyzed ovarian cancer research by assessing the country-specific publication output associated with this topic. DEMP analysis provided the visualization of computed geospatial information regarding our findings, which is a unique strength of this study. Researchers from different countries and continents can benefit from our data since they provide objective insights about the status of ovarian cancer research in their homeland or a specific country of interest. In particular, they are able to plan future research initiatives and collaborations tailored to meet the identified needs. Further, representatives of funding institution can use the presented results for the strategic allocation of resources according to obvious shortcomings. A limitation of this study is linked to the preference of the WoS to index mostly English publications. This translates into an underrepresentation of non-English items and an underestimation of the total article number, which seems to skew our findings. Since high quality research is mostly published in English journals and the WoS catalogs 90% of cited and 80% of published items related to a specific topic [57], our search identified the majority of relevant published items linked to ovarian cancer. Hence, the bias can be considered as limited. Overall, we assessed three types of bibliometric indicators gauging the publication activity on ovarian cancer: Quantitative aspects to measure the productivity of the research community, performance indicators to reflect the quality of scientific output and structural indicators to visualize the interconnectedness of research [58]. Limitations are linked to the evaluation of “qualitative” citation parameters. It is generally accepted that high citation numbers reflect outstanding scientific recognition. This relationship might be skewed due to the Matthew effect: Scientists of acknowledged standing will be cited more than little-known authors, and the citation count of their papers will increase disproportionally after their publications gained some initial popularity [59]. Also, the use of performance indicators (e.g. the citation rate) only helps gauge the quality of published research. Because citation habits and dynamics are highly variable in the investigated fields of research, all variables based on citation frequency are problematic and rather mirror the recognition of fellow scientists than truly reflect quality [59].

## Conclusions

This density-equalizing mapping study represents the first concise analysis of the global ovarian cancer research architecture and illustrates the benefits of scientometrics to assess research output in a standardized way. Our study identifies historically interesting aspects in the research dynamics and relates these to landmark publications in the field. The identification of key manuscripts, subject areas as well as journals with high publication and citation rates guides individual reading and the future direction of scientific endeavors. Also, our observations highlight the outstanding importance of collaborative networks—such as OCAC—that are able to produce high quality research and apply for grant funding successfully in a joint effort. Further, lower-resource countries with a high disease burden in their population should be included in collaborative networks leading to mutual benefits due to the exchange of samples, epidemiologic data, ideas and gained knowledge.

## Additional files

**Additional file 1.** The glossary describes important terms used in this manuscript.

**Additional file 2: Figure S1**. Density equalizing map of global ovarian cancer incidence numbers. Map depicts the absolute incidence numbers of ovarian cancer of the 25 countries having published more than 100 items on ovarian cancer.

**Additional file 3: Table S1**. Most publishing journals with number of articles, number of citations and citation rate.

**Additional file 4: Figure S2**. Number of articles and citation rate of the most publishing journals regarding ovarian cancer.

**Additional file 5: Table S2**. Most cited articles with country of origin, number of citations and journal.

**Additional file 6: Figure S3**. Subject area analysis of ovarian cancer research. A) Number of articles and citations per subject category. B) Relative proportions of the most assigned subject areas in most active countries.

## Abbreviations

DEMP: density equalizing map projections
CR: citation rate
CIA: Central Intelligence Agency
GDP: gross domestic product
HI: Hirsch-index
NewQIS: New quality and quantity indices in science
NCI: National Cancer Institute
OCAC: Ovarian Cancer Association Consortium
UK: United Kingdom
USA: United States of America
WoS: Web of Science
