# Supplementary material for: Ovarian cancer: density equalizing mapping of the global research architecture
Source: Int J Health Geogr. 2017 Jan 13;16:3. doi: 10.1186/s12942-016-0076-2 (PMC5237222; doi:10.1186/s12942-016-0076-2)
Supplement: Supplementary file 1 — Additional file 1. The glossary describes important terms used in this manuscript. [file 12942_2016_76_MOESM1_ESM.docx]

**Density equalizing mapping projections (DEMP)** - a sophisticated visualization technique illustrating benchmarking processes by the creation of distorted world maps that are resized and distorted in proportion to the selected assessment variables.

**Scientometric -** bibliometric approach that examines the quantity, quality and interconnectivity of the scientific output related to one specific topic in a chronological and geographical context.

**New Quality and Quantity Indices in Science (NewQIS) platform** – data collection platform that was funded in 2005 by a multidisciplinary team and is exclusively established at the Goethe University, Frankfurt. It provides a systematic and reliable approach to collect and evaluate data related to research productivity based on a validated protocol.

**Hirsch Index** - developed by Jorge Hirsch in 2005 to represent the recognition of an author’s research performance within the scientific community based on the assumption that articles with higher scientific quality get cited more often. For example, a Hirsch Index of 10 means that 10 papers produced by a specific author have been cited at least 10 times each.

**Modified Hirsch index** - a semi-qualitative proxy measure that is was adapted to evaluate the productivity of single countries particularly for ovarian cancer research in regards to the quantity of the citations, which represents the recognition within global the scientific community.

**Citation Rate** - describes the number of all citations per total ovarian cancer publication item to approximate the quality of a publication. (self citation)

**Q1** (ovarian cancer-related publications per GDP per capita in 1000 US-$) - a calculated ratio that describes country-specific contributions to ovarian cancer research in regards to their economic resources as represented by the gross domestic product (GDP) per capita.

**Q2** (ovarian cancer-specific articles per 1000 billion US-$ GDP) - a calculated ratio that describes country-specific research productivity in regards to their economic resources as represented by to the total economic power index GDP per 1000 billion US-$.

**Q3** (publications/citizen) - a calculated ratio that that describes country-specific research productivity in regards to manpower as represented by the population size.
