# Supplementary figures and images for: Ovarian cancer: density equalizing mapping of the global research architecture
Source: Int J Health Geogr. 2017 Jan 13;16:3. doi: 10.1186/s12942-016-0076-2 (PMC5237222; doi:10.1186/s12942-016-0076-2)

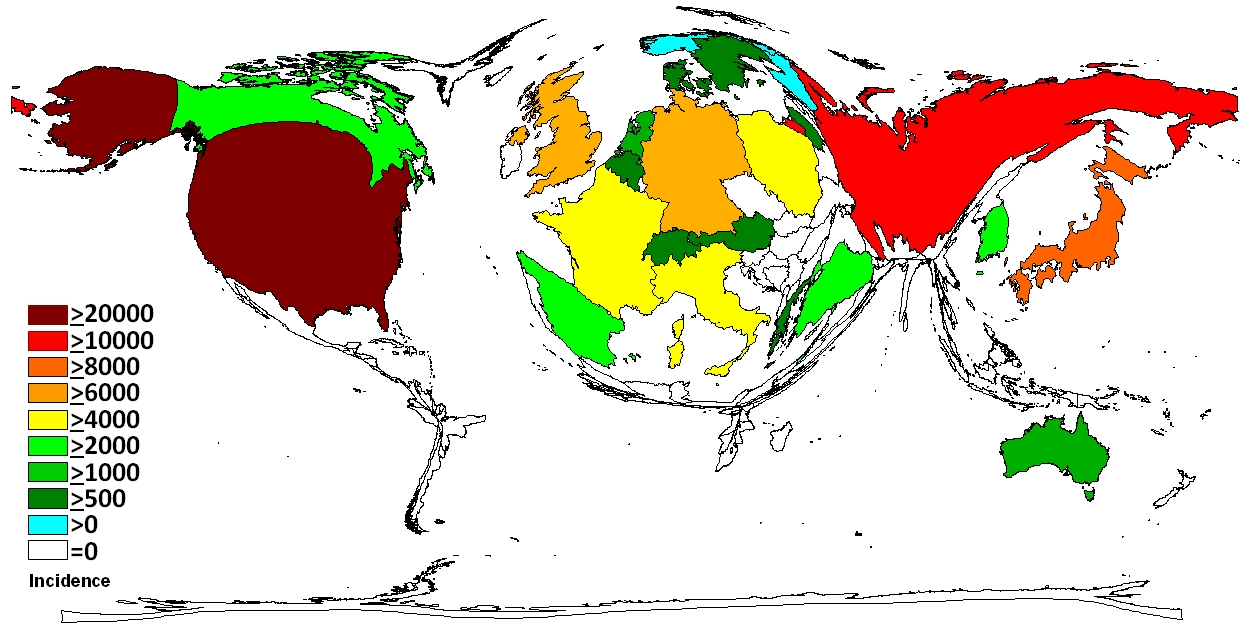

Supplement: Supplementary file 2 — Additional file 2: Figure S1. Density equalizing map of global ovarian cancer incidence numbers. Map depicts the absolute incidence numbers of ovarian cancer of the 25 countries having published more than 100 items on ovarian cancer. [file 12942_2016_76_MOESM2_ESM.jpg]

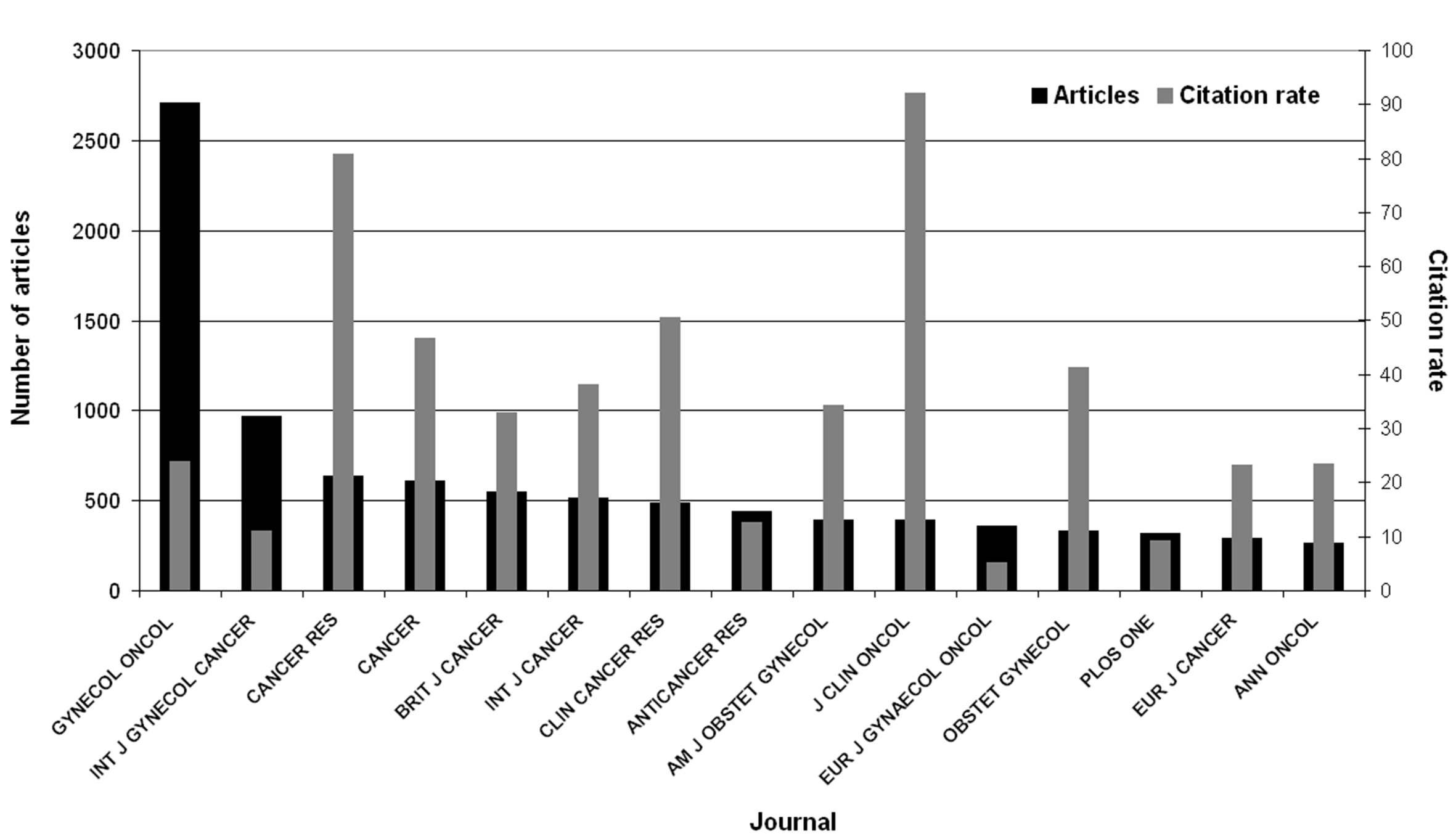

Supplement: Supplementary file 4 — Additional file 4: Figure S2. Number of articles and citation rate of the most publishing journals regarding ovarian cancer. [file 12942_2016_76_MOESM4_ESM.jpg]

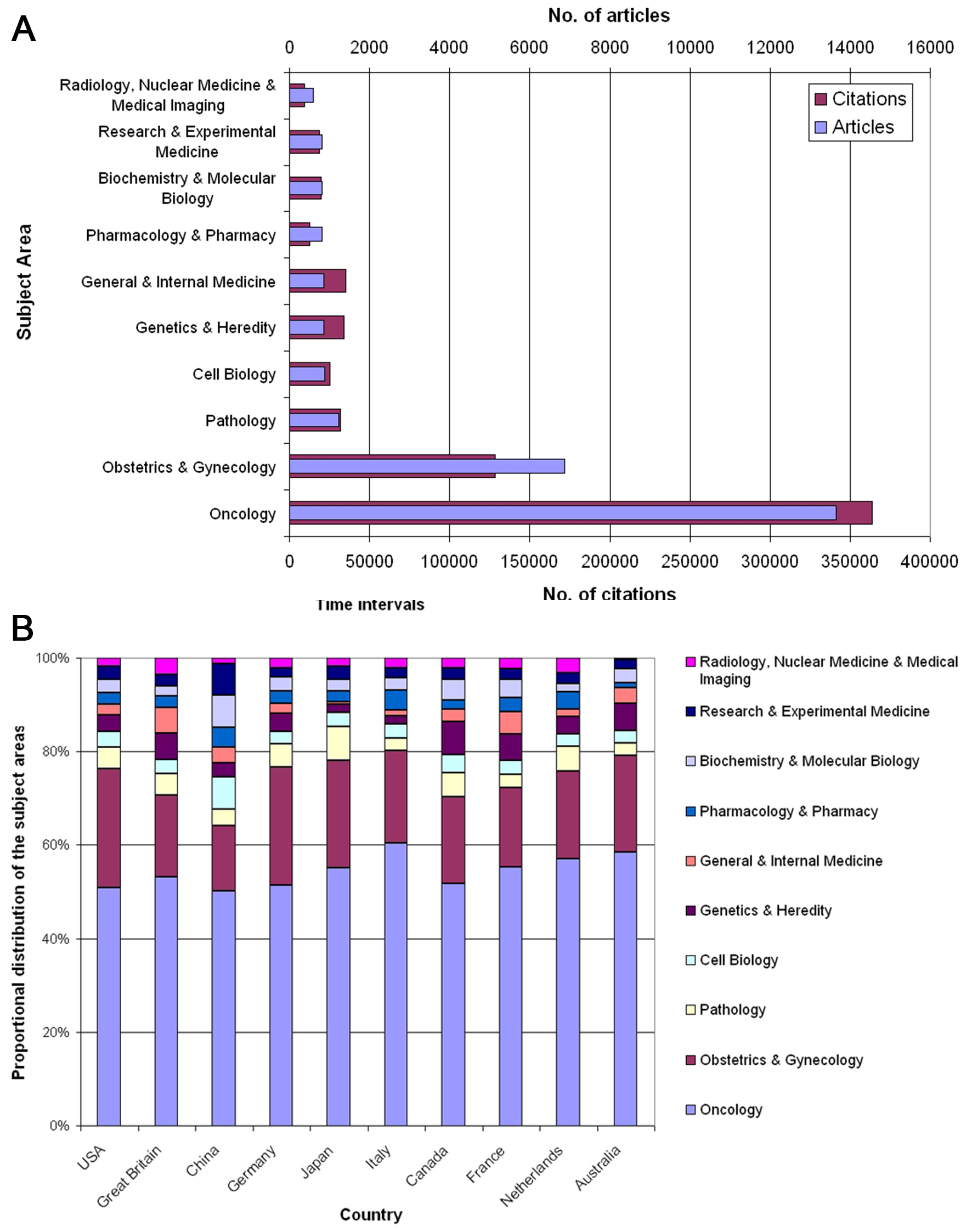

Supplement: Supplementary file 6 — Additional file 6: Figure S3. Subject area analysis of ovarian cancer research. A) Number of articles and citations per subject category. B) Relative proportions of the most assigned subject areas in most active countries. [file 12942_2016_76_MOESM6_ESM.jpg]
