# Supplementary material for: Ovarian cancer: density equalizing mapping of the global research architecture
Source: Int J Health Geogr. 2017 Jan 13;16:3. doi: 10.1186/s12942-016-0076-2 (PMC5237222; doi:10.1186/s12942-016-0076-2)
Supplement: Supplementary file 3 — Additional file 3: Table S1. Most publishing journals with number of articles, number of citations and citation rate. [file 12942_2016_76_MOESM3_ESM.docx]

| Journal | No. of Publications | No. of Citations | Citation rate |
| --- | --- | --- | --- |
| GYNECOL ONCOL | 2710 | 64795 | 23,91 |
| INT J GYNECOL CANCER | 968 | 10707 | 11,06 |
| CANCER RES | 637 | 51479 | 80,81 |
| CANCER | 607 | 28402 | 46,79 |
| BRIT J CANCER | 550 | 18105 | 32,92 |
| INT J CANCER | 516 | 19661 | 38,10 |
| CLIN CANCER RES | 488 | 24682 | 50,58 |
| ANTICANCER RES | 442 | 5558 | 12,57 |
| AM J OBSTET GYNECOL | 396 | 13574 | 34,28 |
| J CLIN ONCOL | 394 | 36303 | 92,14 |
| EUR J GYNAECOL ONCOL | 362 | 1854 | 5,12 |
| OBSTET GYNECOL | 331 | 13679 | 41,33 |
| PLOS ONE | 316 | 2955 | 9,35 |
| EUR J CANCER | 293 | 6828 | 23,30 |
| ANN ONCOL | 266 | 6246 | 23,48 |
