# Supplementary material for: Ovarian cancer: density equalizing mapping of the global research architecture
Source: Int J Health Geogr. 2017 Jan 13;16:3. doi: 10.1186/s12942-016-0076-2 (PMC5237222; doi:10.1186/s12942-016-0076-2)
Supplement: Supplementary file 5 — Additional file 5: Table S2. Most cited articles with country of origin, number of citations and journal. [file 12942_2016_76_MOESM5_ESM.docx]

| Title | Publication Year | Country | Citations | Journal |
| --- | --- | --- | --- | --- |
| Studies of the HER-2/NEU proto-oncogene in human-breast and ovarian-cancer  *(Slamon et al.)* | 1989 | USA, Canada | 4985 | SCIENCE |
| A strong candidate for the breast and ovarian-cancer susceptibility gene BRCA1  *(Miki et al.)* | 1994 | USA, Canada | 3688 | SCIENCE |
| Specific recruitment of regulatory T cells in ovarian carcinoma fosters immune privilege and predicts reduced survival  *(Curiel et al.)* | 2004 | USA | 2105 | NATURE MED |
| Use of proteomic patterns in serum to identify ovarian cancer  *(Petricoin et al.)* | 2002 | USA | 1977 | LANCET |
| Cyclophosphamide and cisplatin compared with paclitaxel and cisplatin in patients with stage III and stage IV ovarian cancer  *(McGuire et al.)* | 1996 | USA | 1956 | N ENGL J MED |
| A radioimmunoassay using a monoclonal-antibody to monitor the course of epithelial ovarian-cancer, *(Bast et al.)* | 1983 | USA | 1627 | N ENGL J MED |
| Average risks of breast and ovarian cancer associated with BRCA1 or BRCA2 mutations detected in case series unselected for family history: A combined analysis of 22 studies, *(Antoniou et al.)* | 2003 | United Kingdom, Canada, USA, Iceland, Australia, Sweden, Italy, China, Hungary, Poland, Finland | 1232 | AM J HUM GENET |
| Reactivity of a monoclonal-antibody with human ovarian-carcinoma  *(Bast et al.)* | 1981 | USA | 1192 | J CLIN INVEST |
| AIB1, a steroid receptor coactivator amplified in breast and ovarian cancer  *(Anzick et al.)* | 1997 | USA, Finland, Switzerland | 1184 | SCIENCE |
| Genetic-linkage analysis in familial breast and ovarian-cancer results from 214 families  *(Easton et al.)* | 1993 | UK | 1072 | AM J HUM GENET |
